# Factors affecting participant recruitment and retention in borderline personality disorder research: a feasibility study

**DOI:** 10.1186/s40814-021-00915-y

**Published:** 2021-09-20

**Authors:** Julia Woo, Hamnah Shahid, Alannah Hillmer, Alamna Abdullah, Sarah Deshpande, Balpreet Panesar, Nitika Sanger, Zena Samaan

**Affiliations:** 1 Department of Psychiatry and Behavioural Neurosciences, McMaster University, 1280 Main Street West, Hamilton, ON Canada; 2 Department of Psychology, Brock University, St. Catharines, ON Canada; 3 Dapartment of Psychology, Neuroscience & Behaviour, McMaster University, Hamilton, ON Canada; 4 Faculty of Health Sciences, McMaster University, Hamilton, ON Canada; 5 Medical Sciences Graduate Program, McMaster University, Hamilton, ON Canada

**Keywords:** Borderline personality disorder, Recruitment, Retention

## Abstract

**Background:**

Previous studies have shown that stigma is a major barrier to participation in psychiatric research and that individuals who participate in psychiatric research may differ clinically and demographically from non-participants. However, few studies have explored research recruitment and retention challenges in the context of personality disorders.

**Aim:**

To provide an analysis of the factors affecting participant recruitment and retention in a study of borderline personality disorder among general psychiatric inpatients.

**Methods:**

Adult inpatients in a tertiary psychiatric hospital were approached about participating in a cross-sectional study of borderline personality disorder. Recruitment rates, retention rates, and reasons for declining participation or withdrawing from the study were collected. Demographic characteristics were compared between participants and non-participants and between patients who remained in the study and those who withdrew.

**Results:**

A total of 71 participants were recruited into the study between January 2018 and March 2020. Recruitment and retention rates were 45% and 70%, respectively. Lack of interest was the most commonly cited reason for non-participation, followed by scheduling conflicts and concerns regarding mental/physical well-being. Age and sex were not predictors of study participation or retention.

**Conclusions:**

More research is needed to explore patients’ perspectives and attitudes towards borderline personality disorder diagnosis and research, determine effects of different recruitment strategies, and identify clinical predictors of recruitment and retention in personality disorder research.

## Key messages regarding feasibility

1. What uncertainties existed regarding the feasibility?

There were uncertainties regarding the barriers to participant recruitment and retention in borderline personality disorder research and potential strategies for improving recruitment and retention.

2) What are the key feasibility findings?

Lack of interest was the most commonly cited reason for non-participation, followed by scheduling conflicts and concerns regarding mental/physical well-being. Age and sex were not predictors of study participation or retention.

3) What are the implications of the feasibility findings for the design of the main study?

The lessons from the feasibility findings were applied to address the common barriers to participant recruitment and retention in the main study design. For instance, strategies were implemented to address the stigma surrounding borderline personality disorder, resolve scheduling issues, and make the study process less stressful and tiring for participants.

## Background

Borderline personality disorder (BPD) is one of the most common comorbidities in psychiatric populations. BPD is found in 10–12% of psychiatric outpatients and 20–22% of inpatients [1]. BPD is also associated with a high burden of disease. About 75% of patients attempt suicide while 5–10% die from suicide [2, 3], making it one of the most lethal psychiatric diagnoses. Longitudinal studies also demonstrate severe and persistent social impairment [3, 4].

Despite the high morbidity and mortality of BPD, it continues to be heavily stigmatized [5, 6]. Negative perceptions of and emotional responses to BPD are common among patients, public, and even healthcare workers [5, 6]. Stigma leads to significant discrimination in the healthcare system, poses a barrier to diagnosis and treatment seeking, disrupts the therapeutic alliance, and hinders treatment adherence and retention [5–7]. Although the effects of stigma on BPD treatment and function have been previously documented, its effects on research are less clear. Previous studies have shown that stigma is a major barrier to participating in research on mental illness in general [8, 9]. This poses a major issue in psychiatric research, as it can reduce external validity of research findings.

In fact, psychiatric research samples do not tend to be representative of the overall patient population [10], and research participants differ significantly from non-participants in terms of illness severity and symptomatology [11, 12]. In addition to participant recruitment, retention rates in research studies can also affect the generalizability and external validity of their findings. For instance, previous psychotherapy trials in BPD have shown that certain clinical variables such as greater illness severity and impulsivity are associated with higher risks of attrition [13]. Better understanding of the factors affecting participant recruitment and retention in BPD research will help contextualize current research findings and allow for more representative samples in future research.

We experienced this issue first-hand in our Transitional Objects in BPD (TOB) project, an observational study exploring a potential association between BPD and the use of transitional objects. We intended to recruit a random sample of general psychiatric inpatients and assess for the association between BPD diagnosis (confirmed through diagnostic interviews) and the use of transitional objects. However, during the recruitment phase of the study, investigators realized that recruitment and retention rates were lower than expected and that many potential participants voiced reluctance about being associated with BPD in any way. Therefore, we decided to conduct a feasibility study to further explore the challenges around participant recruitment and retention.

Overall, this was an exploratory, observational study that was derived from the parent study (TOB study), to help address the challenges we encountered during participant recruitment and data collection. Our primary aim was to identify overall participant recruitment and retention rates as well as the most commonly cited reasons for non-participation and attrition in the TOB study. We hypothesized that stigma around BPD would be a key contributor to non-participation and attrition in the study.

The secondary aim was to look for associations between demographic variables (age and sex), clinical variables (BPD symptom severity), and participant recruitment or retention. While age and sex have not been associated with psychotherapy trial completion in individuals with BPD [13], no study so far has assessed the relationships between age/ sex and participant recruitment and retention in observational BPD research. Similarly, greater illness severity is predictive of psychotherapy trial attrition among patients with BPD [13], but it is unclear if this also extends to observational research in BPD. Therefore, as part of our secondary analysis, we investigated the influence of age, sex, and BPD symptoms on participant recruitment/ retention.

## Methods

This study was approved by the Hamilton Integrated Research Ethics Board (#3786).

### Setting

As previously mentioned, this was a feasibility study built within the TOB study. TOB participants were recruited from inpatient units of a tertiary psychiatric hospital in Canada. The average length of stay for psychiatric inpatients at this facility is about 4 weeks.

### Inclusion and exclusion criteria

Inclusion criteria included 18 years of age or older, ability to provide written informed consent, and psychiatric inpatient at time of recruitment. Participants were excluded if there were English language barriers or if they were about to be discharged from the hospital in the next few days. No restrictions were placed on the type of existing psychiatric diagnoses in order to improve the generalizability and external validity of the sample. Individuals did not need to have a diagnosis of BPD to participate in the study, as we were looking for a relationship between BPD diagnosis and transitional objects and therefore required both BPD and non-BPD participants within our sample.

### Recruitment

Investigators approached hospital staff in various inpatient units to inquire about patients who may be suitable for the study. After obtaining permission from the patients, staff-recommended patients were approached by the investigators to discuss potential participation in the study. Participants could be recruited into the study at any time during their admission, as long as they consented to being approached by the research team. The study purpose, protocol, and potential benefits and harms were explained to potential participants. Written and verbal informed consent was obtained from each participant. Participants were not provided with any form of reimbursements (monetary or otherwise) for participation.

TOB study recruitment began in January 2018 and ended in March 2020. Initial recruitment goal was 60 participants (30 in the BPD group and 30 in controls). This target was based on the rule of thumb that generally, 10 participants are needed per predictor variable to create a stable statistical model [14]. Therefore, to account for three predictive variables (psychiatric disorder, age, and sex), we hoped to acquire a minimum of 30 participants in each group. Recruitment was terminated in March 2020 due to COVID-19 related restrictions. At this time, 71 participants had been recruited into the study and 50 remained in the study; which was below our target of 60. However, due to the ongoing restrictions placed by the pandemic, new pandemic-related stressors and their impact on the study population, as well as changes in the admission criteria, a decision was made to terminate study recruitment at this time.

Unfortunately, at the beginning of the study, no record was kept of the number of individuals approached, the number of individuals who declined participation, and common reasons for declining. Anecdotally, the consensus felt among the study coordinators was that recruitment rates were low and that a significant number of approached patients were declining to participate. Having recognized the issues with recruitment, our team began to maintain a record of the number of individuals approached for the study in June of 2019.

### Procedures

Participation in the TOB study consisted of the following: (1) completion of self-report questionnaires, including the Borderline Symptom List 23 (BSL-23) and (2) a 2-h interview with a study investigator, including the Structured Clinical Interview for DSM 4 Personality Disorders (SCID-4-PD), which took place about seven days following recruitment.

Participant recruitment and interviews were completed by trained student researchers (undergraduate or graduate students in psychology, health research methodology, or a related field). All interviewers were trained on the proper conduct and scoring of the SCID by a clinical psychologist. The student researchers completed practice interviews on one another and conducted an interview under the observation of a clinical psychologist, before they began to perform assessments independently.

### Outcome measures

In this feasibility study, we report on the data related to TOB study recruitment and retention. The outcomes of the TOB study will be reported elsewhere.

The primary outcomes of interest in this feasibility study were as follows: (1) recruitment rate, (2) retention rate, and (3) common reasons for declining to participate in or withdrawing from the study. Recruitment rate was defined as the proportion of approached potential participants who consented to participate in the study. Retention rate was defined as the proportion of recruited participants who did not withdraw consent from the study. We analyzed common reasons for declining to participate in or withdrawing from the study, by categorizing the reasons provided by participants based on common themes such as a discharge from hospital, scheduling issues, or a lack of interest.

During recruitment, age and sex information were collected verbally from each patient who was approached about participating in the study. Age and sex were chosen as potential predictor variables as they were the most convenient and feasible to collect from individuals who declined to participate in the study. Potential participants were also asked verbally to provide reasons for declining to participate in the study. Similarly, participants who withdrew consent from the study were asked to provide reasons for doing so. To minimize bias, those who declined to participate or withdrew consent were simply asked an open question (phrased as, “May I ask why you are declining to participate?” or “How come you are withdrawing from the study?”), instead of being prompted with potential reasons.

As part of the secondary analysis, we assessed the relationships between recruitment/ retention and age, sex, and the presence of BPD symptoms. The BSL-23 was used to screen for symptoms of BPD [15]. An average score greater than two (on a scale from zero to four) was used as the cutoff value [15].

### Data analysis

Recruitment and retention rates as well as key demographic and clinical variables of the sample were reported using descriptive statistics (N and percentages). Similarly, we reported the proportion of participants who identified each reason for non-participation or attrition in the study.

As part of the secondary analysis, mean age and sex were compared between participants and non-participants. Mean age, sex, average BSL-23 scores, and proportion of those who screened positive for BPD on the BSL-23 were compared between participants who remained in the study and those who withdrew. Shapiro-Wilk Test was used to test for normality. Two-tailed, independent samples *t* test was used to compare normally distributed continuous variables, and Mann-Whitney *U* test was used for non-normally distributed continuous variables. Pearson *χ*^2^ test were used for comparing categorical variables. The Statistical Package for Social Sciences Version 21 was used. Statistical significance was set at *α* = 0.05.

## Results

### Sample characteristics

Seventy-one participants were recruited into the TOB study. Demographic and clinical characteristics of the recruited participants are shown in Table 1. Fifty-eight of the participants completed the BSL-23. Of those, 31 (53%) screened positive for BPD in the BSL-23. The prevalence of BPD based on the SCID in the study population was 29%.

**Table 1:** Demographic and clinical characteristics of recruited participants

Characteristic	
Mean age	39.3 ± 14.4
Sex	
Female	44 (62%)
Male	27 (38%)
Ethnicity (*N* = 70)	
European	53 (76%)
Other/mixed	8 (11%)
East/South Asian	6 (9%)
African	3 (4%)
Reasons for admission (*N* = 54)^a^	
Suicidal ideation/attempt	24 (44%)
Depression	18 (33%)
Unclear	7 (13%)
Aggression/harm to others	3 (6%)
Mania/mixed mood episode	3 (6%)
Psychosis	3 (6%)
Substance use disorder	2 (4%)
Other	2 (4%)
Pre-existing psychiatric diagnoses (*N* = 54)^a^	
Depression	21 (39%)
Bipolar disorder	17 (31%)
Anxiety disorder	12 (22%)
Borderline personality disorder (BPD)	12 (22%)
Post-traumatic stress disorder	9 (17%)
ADHD	6 (11%)
Schizophrenia/schizoaffective disorder	6 (11%)
No previous diagnosis	6 (11%)
Obsessive compulsive disorder	5 (9%)
Eating disorder	2 (4%)
Unknown	1 (2%)
Screens positive for BPD (*N* = 58)^b^	31 (53%)
Meets diagnostic criteria for BPD (*N* = 38)^c^	11 (29%)

^a^Participants often had more than one reason for admission and more than one pre-existing diagnosis

^b^An average score greater than two (on a scale from zero to four) on the Borderline Symptom List 23

^c^Based on the Structured Clinical Interview for DSM 4 Axis II Personality Disorders

### Recruitment and retention rates

As previously mentioned, between January 2018 and May 2019, we were not keeping track of the number of individuals who had been approached about participating in the study. Beginning in June 2019, we began to maintain a record of the number of patients who were approached about study participation, the age and gender of each patient who was approached, as well as patient-reported reasons for declining to participate.

Between January 2018 and May 2019, 34 participants were recruited into the TOB study. Between June 2019 and March 2020, 82 potential participants were approached regarding the study. 37 consented (45%) and 45 declined to participate (55%). In total, 71 participants were recruited into the study between January 2018 and March 2020. Of those, 21 (30%) withdrew consent and 50 (70%) remained in the study.

### Reasons for non-participation and attrition

Table 2 shows the reasons provided for declining to participate in and withdrawing consent from the study. As shown in Table 2, “not interested” was the most common reason provided for non-participation (*N* = 24, 53%). When asked further about why they were not interested, the most common response was, “I do not have borderline.” One particular patient adamantly denied having any mental illness.

**Table 2:** Reasons for declining to participate in the study or for withdrawing consent from the study

Reason for declining (*N* = 45)	*N*
“Not interested”	24 (53%)
Imminent discharge	12 (27%)
Already enrolled in study	4 (9%)
Too unwell to participate	2 (4%)
Interview process is too long	2 (4%)
Not reported	1 (2%)
Reason for withdrawing (*N* = 21)	*N*
No reason provided	9 (43%)
Discharged from hospital	6 (29%)
Interview process too upsetting/ tiring	4 (19%)
Too unwell to continue	2 (10%)

In addition, six individuals declined participation or withdrew because they found the interview process either too long or too emotionally exhausting. In particular, two participants found questions in the structured interviews too triggering and thus asked to terminate the interview. Similarly, four individuals reported feeling too unwell to engage meaningfully in the study.

Another challenge encountered in the study was participants forgetting the date and time of their interview. There were also scheduling conflicts with other activities such as off-ward privileges, medical imaging, group sessions, or assessments with other healthcare providers. Every effort was made to reschedule the interview and to contact the participants again. However, at times, the study personnel were unable to reach the participant on multiple occasions, and thus four patients were discharged before they could be seen. Among the nine participants who did not provide a reason for withdrawing consent, many of them had also failed to attend their scheduled interviews on multiple occasions prior to leaving the study.

### Secondary analysis

Table 3 compares demographic and clinical characteristics between participants and non-participants, as well as between patients who remained in the study and those who withdrew. There were no statistically significant differences in age (recruitment: *U* = 1901, *p* = 0.086; retention: *U* = 435, *p* = 0.257) or sex between groups (recruitment: *χ*^2^ = 0.072, *p* = 0.788; retention: *χ*^2^ = 0.295, *p* = 0.587). In comparing participants who remained in the study and those who withdrew consent, there was no statistically significant difference in the average BSL-23 score (*U* = 232, *p* = 0.869) or the proportion of individuals who screened positive for BPD on the BSL-23 (*χ*^2^ = 0.058, *p* = 0.810).

**Table 3:** Demographic and clinical comparisons

	Consented (*N* = 71)	Declined (*N* = 45)	Statistic
Mean age	39.3 ± 14.4	43.6 ± 13.1	*U* = 1901, *p* = 0.086
Sex			
Female	44 (62%)	29 (64%)	*χ*^2^ = 0.072, *p* = 0.788
Male	27 (38%)	16 (36%)	
	Remained in study (*N* = 50)	Withdrew from study (*N* = 21)	Statistic
Mean age	40.8 ± 15.0	35.8 ± 12.2	*U* = 435, *p* = 0.257
Sex			
Female	32 (64%)	12 (57%)	*χ*^2^ = 0.295, *p* = 0.587
Male	18 (36%)	9 (43%)	
Average BSL-23 score^a^	2.03 ± 1.08, *N* = 48	1.98 ± 0.98, *N* = 10	*U* = 232, *p* = 0.869
Screens positive for BPD^b^	26 (54%), *N* = 48	5 (50%), *N* = 10	*χ*^2^ = 0.058, *p* = 0.810

^a^BSL-23: Borderline Symptom List 23

^b^An average score greater than two (on a scale from zero to four) on the BSL-23

## Discussion

### Recruitment and retention

In our sample, 45% of psychiatric inpatients who were approached were recruited into the TOB study, and 70% of the recruited participants remained in the study.

Few studies have reported research recruitment rates in BPD population in the past. A RCT of dialectical behavioral therapy in women with BPD in Netherlands reported a recruitment rate of 70% [16]. Our retention rate was consistent with a meta-analysis of psychotherapy trials for BPD, which reported an overall retention rate of 71–75% [13].

There are several potential reasons behind the differences in recruitment rates between our study (45%) and the past RCT in BPD (70%) [16]. One potential reason is that our sample consisted of general psychiatric inpatients, whereas the aforementioned studies examined individuals with BPD. Moreover, our participants were recruited from an inpatient setting, whereas the previous studies on recruitment and retention rates focused on outpatients. The inpatient setting may have made it easier to both recruit and retain participants in our study, by removing potential barriers to participation such as access to transportation and conflicts with work or other commitments. Moreover, the TOB study was observational in nature and mainly involved the completion of self-report questionnaires and a 2-h interview, whereas the aforementioned psychotherapy trials for BPD were interventional in nature. This may have meant that our study presented a relatively low barrier to participation, as it did not involve an extensive time commitment or partaking in a novel study intervention. On the other hand, this could have made our study less enticing for some patients, as participants were not given access to treatment that they would not have been able to access otherwise.

### Reasons for non-participation or withdrawing consent

Overall, “not interested” was the most common reason provided for declining to participate in the TOB study. Although this is very broad explanation that can encompass many different reasons, it seemed to frequently stem from the stigma related to BPD, which was consistent with our initial hypothesis and experiences during study recruitment. Potential participants frequently expressed discomfort about being associated with BPD and were quick to emphasize that they do not have a diagnosis of BPD. This is consistent with previous findings that BPD is by far one of the most stigmatized psychiatric disorders, even within the mental health system itself [5, 6]. In fact, attitudes and behaviors of mental health workers towards BPD are even more negative than other illnesses such as schizophrenia and mood disorders [17–19]. Individuals with BPD are often perceived as “annoying” and “undeserving of sympathy” by the public [5, 6]. As a result, BPD patients experience greater levels of existential shame and self-stigma compared to those with other mental illnesses [20] and report significant discriminatory experiences [21]. Stigma is also a common barrier to participation in psychiatric studies in general. In a systematic review of this topic, Woodall et al. reported stigma and lack of acceptance of diagnosis as two of the most common barriers to research recruitment among patients with mental illness [8].

Another potential reason behind the lack of interest in participation was that we were unable to provide participants with monetary or other forms of reimbursement for their time and effort. Similarly, the study process was quite long and involved 2 h of psychological interviewing and administration of several standardized questionnaires—a process that can feel intrusive and tiring for many patients.

In terms of study retention, there were some logistical challenges around scheduling. This issue is by no means specific to BPD or psychiatry, and is commonly encountered in all clinical trials [8, 9, 22]. In fact, the inpatient setting often allows for easier scheduling and access to participants compared to outpatient clinics. Regardless, 29–72% of the participants withdrew from our study either directly or partially due to scheduling issues.

Another major factor in recruitment and retention was the well-being of the patients. Two patients were unable to participate meaningfully in the study due to the poor state of their physical or mental health. The fact that four participants withdrew consent because they found the study process too upsetting or tiring is perhaps unsurprising, given that the study involved lengthy interviews with highly personal questions. This may also reflect the fluctuating course of most mental illnesses. In fact, illness severity and fear of exacerbating illness were two of the largest themes that arose in the systematic review by Woodall et al. [8].

### Relationships between recruitment, retention, and demographic or clinical variables

Age and sex were not predictors of recruitment or retention. This is similar with previous clinical trials on BPD, which reported no significant association between psychotherapy completion and sociodemographic factors [13]. However, this differs from research in psychotic disorders which have found younger age to be linked with great rates of recruitment and retention [11, 12]. The differences in findings may be due in part to the differences in study populations (general psychiatric inpatients vs patients with psychotic disorders) as well as the overall study designs (clinical vs observational). The clinical diversity of our patient population (i.e., a population consisting of numerous differing psychiatric diagnoses) also may have made it difficult to identify overarching sociodemographic predictors of recruitment and retention in our sample.

Similarly, screening positive for BPD or having greater severity of BPD symptoms was not associated with recruitment or retention. This is in contrary to past studies which found illness severity to be associated with higher risks of attrition, in patients with BPD [13] as well as general psychiatric inpatients [23]. One possibility behind this is that we assessed BPD symptoms in a sample of general psychiatric inpatients; given that many individuals in our sample did not have BPD, and given the diversity of psychiatric comorbidities in our sample, overall level of functioning may have been a more accurate predictor of recruitment and retention.

### Strategies used to improve recruitment and retention

Having identified these challenges, throughout the TOB study we began to implement some strategies to improve recruitment and retention. To target the stigma surrounding BPD, the need for controls in the study was emphasized; being part of the study did not necessarily mean that the participant had BPD, as the aim of the study was to explore BPD characteristics in a random sample of psychiatric inpatients. Initially, the phrasing we had used during participant recruitment would be, “We are conducting a study on the relationship between BPD and comfort objects.” Over time, we added more clarification to help highlight the fact that we were not exclusively recruiting individuals with BPD: “Although the study is aimed to investigate BPD and comfort objects, we are looking for anyone on the unit who would be interested in participating. You do not need to have BPD to participate.”

Notably, a similar strategy was utilized in a RCT of a 1-day CBT workshop for individuals with depression [24]. Brown et al. identified that re-labeling the study intervention from “depression workshops” to “self-confidence workshops” resulted in a marked increase in recruitment, although they did not compare recruitment rates before and after this change was implemented [24]. This re-labeling was based on the close association between depression and low self-esteem. As well, the investigators felt that they were able to make the study more understandable and acceptable to the public by utilizing a more colloquial and non-diagnostic term (self-confidence) as opposed to a diagnostic title (depression). The authors discussed that by using non-diagnostic “lay” titles, studies may be able to reach out to those who do not perceive a need for treatment, have undiagnosed mental illness, or might be reluctant to engage in treatment due to the stigma surrounding psychiatric labels [24].

Around June 2019, we also began to employ more investigators for participant recruitment and assessments (two investigators at a time compared to one). As well, around this time we began to visit the inpatient units more frequently (once daily compared to one to two times per week). We also increased the number of inpatient units we were visiting for participant recruitment; whereas we had only been visiting the mood disorders unit previously, we began to recruit participants from the acute, concurrent disorders, and schizophrenia units as well. Moreover, in collaboration with the inpatient unit staff, we began to identify times of the day that may be ideal for participant recruitment and interviews, based on each unit’s schedule and daily activities. For instance, efforts were made to avoid visiting the units for recruitment during mealtimes or medication administration. We also created and maintained a running list of inpatients who may potentially be eligible for the study, based on input from the inpatient unit staff.

With regard to the scheduling challenges, the study investigators communicated more closely with inpatient unit staff to avoid any scheduling conflicts. Written and verbal reminders were also used. Lastly, to make the process less tiring for participants, we offered to perform the interview in multiple shorter segments. Participants were reminded that they could pause or leave the interview at any time.

As these strategies were gradually implemented over time (starting in around June 2019) and not in a structured manner, we were unable to analyze their effects on recruitment and retention rates. In the 16-month period before we began to track recruitment rates (between January 2018 and May 2019), 34 participants were recruited; on the other hand, in the 9-month period after we began to track recruitment rates, we recruited 37 participants (June 2019–March 2020). This apparent increase in recruitment speed may have been partially due to the strategies we described above, although this is impossible to ascertain for certain as we do not know how many individuals were approached about the study between January 2018 and May 2019. Generally, it was felt that the strategies used to target stigma were not very effective. Although patients were reassured that participation in the study did not mean that they had BPD, patients still continued to express discomfort about being associated with BPD in any way. Considering the prevalent and deep-rooted nature of the stigma surrounding BPD, there is only so much that can be done in individual studies to address it. There is a need for more concerted, large-scale efforts to address the stigma surrounding BPD, such as public awareness campaigns and psychoeducation of diagnosed patients [6].

A recent systematic review reported on various recruitment strategies used to overcome age-, gender-, and race-related barriers in psychiatric research such as travel support, avoiding stigmatizing language, and better education about the purpose and nature of the investigation [8]. However, very few of these strategies have been formally evaluated and thus their relative efficacies are unclear [8]. In a more recent systematic review, Liu et al. found 11 studies on recruitment and two studies on retention strategies in mental health trials [25]. Recruitment by clinical research staff and non-web-based advertisements were found to be effective in improving recruitment, while the use of abridged questionnaires and regular reminders were helpful in retention. Financial incentives were effective in improving both [25]. Outside of psychiatry, stakeholders’ advisories, personalized update letters, and educational materials have been reported as successful recruitment and retention strategies in clinical trials [26, 27].

Another important factor to consider is that individuals from lower socioeconomic status (SES) or minority backgrounds often disproportionately face barriers to research recruitment and retention. Low health literacy; difficulty taking time away from work; poor access transportation; lack of a reliable phone or other means to schedule follow-up appointments; and negative experiences with the healthcare system at large may all create additional barriers to research participation among those facing precarious housing, poverty, unemployment, illiteracy, or low education [8, 28]. Moreover, individuals from ethnic minority backgrounds tend to have more mistrust towards medical research at large [8]. In fact, the National Survey of American Life found that refusal to participate in mental health research was higher among African Caribbean participants, driven in part by fears of possible questions about immigration status [29]. Language barriers and cultural stigma surrounding mental illness are also additional barriers among some ethnic groups [8]. These trends are particularly concerning given the fact that many psychiatric diagnoses (including BPD) tend to be overrepresented in ethnic minorities and low SES populations and that such groups tend to face worse outcomes compared to their Caucasian, high SES counterparts [30, 31].

These issues related to inequity and social determinants of health are further exacerbated by the COVID-19 pandemic. As mentioned previously, our recruitment was halted due to pandemic-related restrictions on research recruitment. There has been mounting evidence that pre-existing socioeconomic barriers and systemic injustices affecting low-income communities, ethnic minorities, individuals with mental illness, and other disadvantaged populations have been amplified by the pandemic [32]. Although the impact on long-term clinical research in psychiatric populations is unclear at this time, it is possible that the pandemic may further exacerbate the barriers to research recruitment and retention going forward.

To overcome the challenges involving the recruitment and retention of individuals from low SES groups, some studies have utilized strategies such as hiring outreach workers who are from the targeted community, collecting alternative contacts for subjects with precarious housing, and providing means of transportation in order to facilitate better engagement and recruitment of ethnic minorities and low SES communities. However, very few of these strategies have been formally evaluated in the psychiatric setting, limiting their generalizability [8]. There is, however, evidence showing that matching recruiters to potential participants based on ethnicity is not effective in improving recruitment of ethnic minorities in psychiatric research [8].

### Limitations

There are several limitations of the current study. Firstly, no data on recruitment rates were collected at the beginning of the study. We only began to collect this information after implementing several recruitment and retention strategies; thus, it is possible that recruitment and retention were lower previously and that the reported values are an underestimate of the study average. Secondly, the strategies were gradually implemented over time and thus their effects on recruitment and retention could not be evaluated. Thirdly, only brief descriptions were provided by participants regarding their reason for declining consent or withdrawing from the study. Fourth, limited data were available on individuals who declined to participate, making it challenging to examine clinical predictors of recruitment. Similarly, we were unable to compare key clinical variables such as illness severity, reason for admission, or psychiatric comorbidities between participants who remained in the study and those who withdrew, as the latter group often did not complete the structured clinical interviews such as the SCID. Fifth, only patients who were deemed to be suitable candidates were approached by the recruiters, introducing sampling bias. The more severely ill and agitated patients were likely left out of the study, thus underestimating the true recruitment and retention rates in this population. Sixth, given that the majority (76%) of our participants were of European origin, the study findings may not be representative of the barriers faced by minorities and those from non-Caucasian backgrounds. Seventh, participants were recruited from a tertiary psychiatric hospital and thus the findings may not be generalizable to outpatient or community-based research settings. Lastly, our sample size was relatively small and thus may not encapsulate all of the most common reasons for non-participation and loss to follow-up in BPD research. Overall, the study findings should be interpreted in the context of the tertiary psychiatric hospital population and limited sample size.

## Conclusions

In our sample of general psychiatric inpatients, recruitment and retention rates were comparable to previous studies on similar populations in outpatient contexts. Recruitment and retention were not associated with age or sex. Lack of interest, scheduling issues, and mental and physical well-being were the biggest barriers to participating and remaining in the study, consistent with findings from previous studies in psychiatric populations. We hope that the findings of the current study will help ensure that future studies are better designed to address the barriers to psychiatric research participation.

The lessons learned from this feasibility study were applied to improve recruitment and retention in the TOB study. As mentioned previously, we were unable to determine the efficacy of these strategies in improving recruitment and retention rates. However, anecdotally it was felt the strategies were not very effective in addressing the deep-rooted stigmas surrounding BPD in a psychiatric population. In this regard, the objectives of the feasibility study were only partially met; while we were able to identify common barriers to recruitment and retention, we were unable to identify effective strategies for addressing them within our study context.

Going forward, qualitative studies would be beneficial in providing an in-depth look at the barriers and facilitators to participation in BPD research. In fact, systematic reviews of recruitment issues in psychiatric research did not identify any studies on personality disorders [8, 25]. Moreover, very few recruitment and retention strategies have been formally evaluated in psychiatric research, and none have been evaluated for personality disorders. Considering that personality disorders may be even more heavily stigmatized than other mental illnesses, it would be worthwhile to explore these issues in the context of personality disorders such as BPD. In particular, it would be important to assess the specific barriers to recruitment and retention faced by ethnic minorities and low SES groups, as well as the strategies that best address those barriers. Lastly, studies have utilized clinical databases to compare demographic and clinical characteristics between research participants and non-participants [11, 12]. Similar approaches could be used for BPD in the future. Doing so will help shed light on whether the current research on BPD is truly representative of the overall patient population and identify potential strategies to improve external validity of BPD research going forward.

## Data Availability

The datasets generated and analyzed during the study are not publicly available to help protect the privacy of the participants. However, they may be available from the corresponding author on reasonable request.

## Abbreviations

BPD: Borderline personality disorder
BSL-23: Borderline symptom list 23
RCT: Randomized controlled trial
SCID: Structured clinical interview for DSM 4
TOB: Transitional objects in borderline personality disorder
